# Genome-Wide Identification and Characterization of miRNAs and Natural Antisense Transcripts Show the Complexity of Gene Regulatory Networks for Secondary Metabolism in *Aristolochia contorta*

**DOI:** 10.3390/ijms25116043

**Published:** 2024-05-30

**Authors:** Wenjing Liang, Yayun Xu, Xinyun Cui, Caili Li, Shanfa Lu

**Affiliations:** 1 State Key Laboratory for Quality Ensurance and Sustainable Use of Dao-di Herbs, Institute of Medicinal Plant Development, Chinese Academy of Medical Sciences & Peking Union Medical College, Beijing 100193, China; 2 Engineering Research Center of Chinese Medicine Resource of Ministry of Education, Institute of Medicinal Plant Development, Chinese Academy of Medical Sciences & Peking Union Medical College, Beijing 100193, China

**Keywords:** *Aristolochia contorta*, aristolochic acid, benzylisoquinoline alkaloid, long non-coding RNA, microRNA, natural antisense transcript

## Abstract

*Aristolochia contorta* Bunge is an academically and medicinally important plant species. It belongs to the magnoliids, with an uncertain phylogenetic position, and is one of the few plant species lacking a whole-genome duplication (WGD) event after the angiosperm-wide WGD. *A. contorta* has been an important traditional Chinese medicine material. Since it contains aristolochic acids (AAs), chemical compounds with nephrotoxity and carcinogenicity, the utilization of this plant has attracted widespread attention. Great efforts are being made to increase its bioactive compounds and reduce or completely remove toxic compounds. MicroRNAs (miRNAs) and natural antisense transcripts (NATs) are two classes of regulators potentially involved in metabolism regulation. Here, we report the identification and characterization of 223 miRNAs and 363 miRNA targets. The identified miRNAs include 51 known miRNAs belonging to 20 families and 172 novel miRNAs belonging to 107 families. A negative correlation between the expression of miRNAs and their targets was observed. In addition, we identified 441 *A. contorta* NATs and 560 NAT-sense transcript (ST) pairs, of which 12 NATs were targets of 13 miRNAs, forming 18 miRNA-NAT-ST modules. Various miRNAs and NATs potentially regulated secondary metabolism through the modes of miRNA-target gene–enzyme genes, NAT-STs, and NAT-miRNA-target gene–enzyme genes, suggesting the complexity of gene regulatory networks in *A. contorta*. The results lay a solid foundation for further manipulating the production of its bioactive and toxic compounds.

## 1. Introduction

*Aristolochia contorta*, belonging to the family Aristolochiaceae in the order Piperales of magnoliids, primarily grows in Liaoning, Jilin, and Heilongjiang provinces of China, but is also distributed in Russia, Japan, and Korea. This plant grows on forest edges, valley sides, and hillsides, favoring warm weather and moist, fertile, sandy, and humus-rich soils. In China, *A. contorta* is well-known for its medicinal properties. The entire plant is beneficial and possesses various medicinal properties, with different parts of the plant serving specific medical purposes. For centuries, *A. contorta* has been extensively used in traditional Chinese medicine (TCM) and has proven successful in treating numerous diseases, such as inflammation reduction, asthma relief, bacteria suppression, and pain management [1]. It is rich in bioactive compounds, such as alkaloids, terpenoids, flavonoids, and others. However, this plant also contains aristolochic acids (AAs), which are nephrotoxic and carcinogenic compounds [2]. To increase the bioactive compounds and reduce the toxic compounds in *A. contorta*, it is crucial to explore the biosynthetic pathways of metabolites and their regulatory mechanisms. Previously, we have sequenced the whole genome of *A. contorta* and identified various key enzyme genes involved in bioactive compound biosynthesis [3,4]; however, their regulatory mechanism remains to be elucidated.

Plant microRNAs (miRNAs) are single-strand, non-coding, and endogenous small RNAs with a length of about 20–22 nt. They act as negative regulators of target genes through direct cleavage or translational inhibition of target transcripts [5]. To generate mature plant miRNAs, primary transcripts, known as *pri-MIRs*, are first transcribed from *MIR* loci under the catalysis of RNA polymerase II (Pol II). Then, the generated *pri-MIRs* are processed into mature miRNAs by the DICER-LIKE endonuclease complex I (DCLl) [6]. Mature miRNAs play important roles in various developmental processes, biotic and abiotic stress responses, secondary metabolite biosynthesis, and DNA methylation [7,8,9,10]. For instance, ptr-miR397a acts as a negative regulator of laccase genes, affecting lignin biosynthesis in *Populus trichocarpa* [11]. Smi-miR858a regulates tanshinone and phenolic acid biosynthesis through cleaving the transcripts of *SmMYB6*, *SmMYB97*, *SmMYB111*, and *SmMYB112* in *Salvia miltiorrhiza* that had its whole genome sequence available [12,13]. Smi-miR159a mediates *SmMYB62*, *SmMYB78*, and *SmMYB80* to regulate phenolic acid biosynthesis in *S. miltiorrhiza* [14]. Ptr-miR397, ptr-miR398, ptr-miR408, and ptr-miR1444 are involved in copper stress responses in *P. trichocarpa* through targeting Cu-responsive genes [15]. Smi-miR12112 mediated polyphenol oxidase genes to regulate phenolic acid biosynthesis and metabolism [16], while lineage-specific Smi-miR7972 regulates DNA methylation through cleaving *SmDML1* transcripts in *S. miltiorrhiza* [17].

Long non-coding RNAs (lncRNAs) are a class of RNA molecules that are longer than 200 nt and do not encode proteins [18]. They play critical functions in gene regulation, chromatin remodeling, cell development, and so on [19]. Based on genomic locations, lncRNAs are generally divided into three types, including long intergenic ncRNAs (lincRNAs), intronic ncRNAs, and non-coding natural antisense transcripts (NATs) [20]. Among them, NATs are transcribed from the opposite strand of a protein-coding gene or another lncRNA and often overlap or are partially complementary to their sense counterparts [21,22,23]. They can interact with their sense partners through base pairing to inhibit the transcription of sense genes or can mediate in chromatin modification [24], where the interaction is complex. One NAT can simultaneously regulate multiple genes nearby but can also affect distant gene loci through long-range chromatin interactions. Additionally, some NATs can function as miRNA decoys. They sequester the small molecules and prevent them from binding to targets, thereby indirectly affecting gene expression [25]. NATs play multifaceted regulatory roles in plants. For instance, in *P. tricornutum*, NATs respond to phosphate (Pi) stress by regulating the expression of its corresponding sense genes involved in cell homeostasis maintenance under pressure [26]. In *Arabidopsis*, *cis*-acting non-coding NATs are found to control seed dormancy [27]. In moso bamboo, *LAC4* and its *cis*-NATs are induced by GA3 and can be involved in lignin accumulation [28]. In *S. miltiorrhiza*, NATs potentially regulate bioactive compound biosynthesis by binding to the sense transcripts encoding pathway enzymes [29].

To the best of our knowledge, miRNAs and NATs have not been characterized and functionally analyzed for *A. contorta* to date, although its whole genome has been sequenced [3]. Through small RNA library sequencing, degradome sequencing, and transcriptome analysis, a total of 223 miRNAs, 363 miRNA targets, 441 NATs, and 560 NAT-sense transcript (ST) pairs have been genome-widely identified from *A. contorta*. Among them, 12 NATs are targets of 13 miRNAs, forming 18 miRNA-NAT-ST modules. Subsequent high-throughput sequence data analysis and qRT-PCR detection has revealed a negative correlation between the expression of miRNAs and their targets. Furthermore, gene regulatory network analysis has shown that miRNAs and NATs can potentially regulate secondary metabolism through the modes of miRNA-target gene–enzyme genes, NAT-STs, and NAT-miRNA-target gene–enzyme genes. It suggests the existence of complex gene regulatory networks for secondary metabolism in *A. contorta*.

## 2. Results and Discussion

### 2.1. High-Throughput Small RNA Sequencing of A. contorta

To perform genome-wide identification of miRNAs in *A. contorta*, we constructed four libraries of small RNAs from roots, stems, leaves, and flowers and sequenced the libraries using the Illumina sequencing platform. A total of 20,509,711, 19,120,948, 27,955,448, and 22,405,111 reads were generated from each library (Table 1). After filtering out low-quality sequences, including junk reads, adaptor sequences, polyA tags, and reads of less than 18 nt and more than 30 nt, a total of 14,699,031, 14,861,342, 19,939,928, and 16,868,747 clean reads were yielded for further analysis. The clean reads were mapped to related databases, such as Rfam (http://rfam.xfam.org/ (accessed on 10 September 2022)) and Repbase (http://www.girinst.org/repbase/ (accessed on 15 October 2022)). Reads derived from mRNA, rRNA, tRNA, snRNA, snoRNA, and repeat sequences were removed. The remaining 13 million small RNAs were used for miRNA prediction.

**Figure 1:**
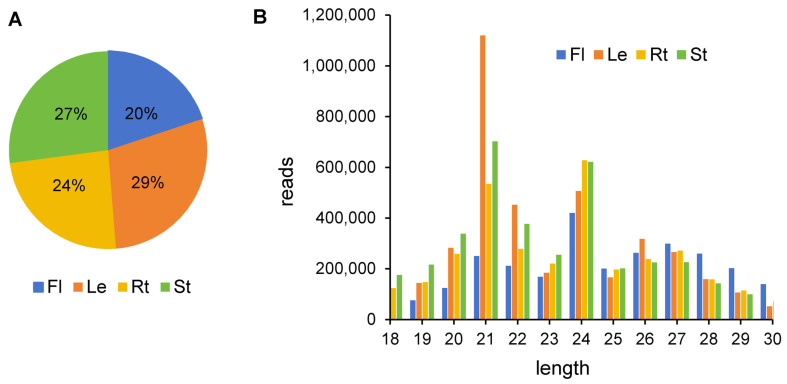
Analysis of *A. contorta*’s small RNA in four tissues. (**A**) Percentage of unique small RNAs from four tissues. (**B**) Size distribution of clean reads.

The number of unique small RNAs from four tissues ranged from two to four million. Leaves had the highest number, whereas flowers had the lowest (Figure 1A). Size distribution analysis showed that the small RNAs identified were primarily 21–24 nt in length (Figure 1B). In leaves and stems, the most abundant length of small RNAs was 21 nt, whereas in roots and flowers, the most abundant small RNAs were 24 nt in length. The results were consistent with previous reports [30].

### 2.2. Identification of 51 Known and 172 Novel miRNAs

In plants, the number of miRNAs varies among species, roughly reflecting their uniqueness in biological evolution and functional diversity. Based on the data in miRbase (22.1) [31] and other publications, *Amborella trichopoda* has 124 miRNAs, *Volvox carteri* has 174 [32], *Larix olgensis* has 78 [33], black pepper has 128 [34], *Arabidopsis thaliana* has 326 miRNAs, *P. trichocarpa* has 352, and *Isodon rubescens* has 348 [35]. This indicates that species at the base of a plant evolutionary tree generally have a smaller number of miRNAs, whereas species at the branching end have a greater number [36,37]. To identify miRNAs from small RNAs of *A. contorta*, clean reads were mapped to the genome assembly of *A. contorta* using psRobot [38] and mirDeep2 [39]. The flanking sequences were extracted and then predicted for secondary structures using RNAfold [40]. Application of the criteria for plant miRNA annotation proposed previously [41] allowed us to identify a total of 223 miRNA precursors (Table S1). The resulting miRNAs were then blast-analyzed with those from other plant species (miRBase 22.1) [42], resulting in the identification of 51 known and 172 novel *A. contorta* miRNAs [42]. *A. contorta* is a species at the base of the evolutionary tree and there is an absence of a whole-genome duplication (WGD) event after the angiosperm-wide WGD [3]. The number of miRNAs identified is consistent with its evolutionary history.

### 2.3. Characterization and Genome-Wide Distribution of A. contorta miRNAs

**Figure 2:**
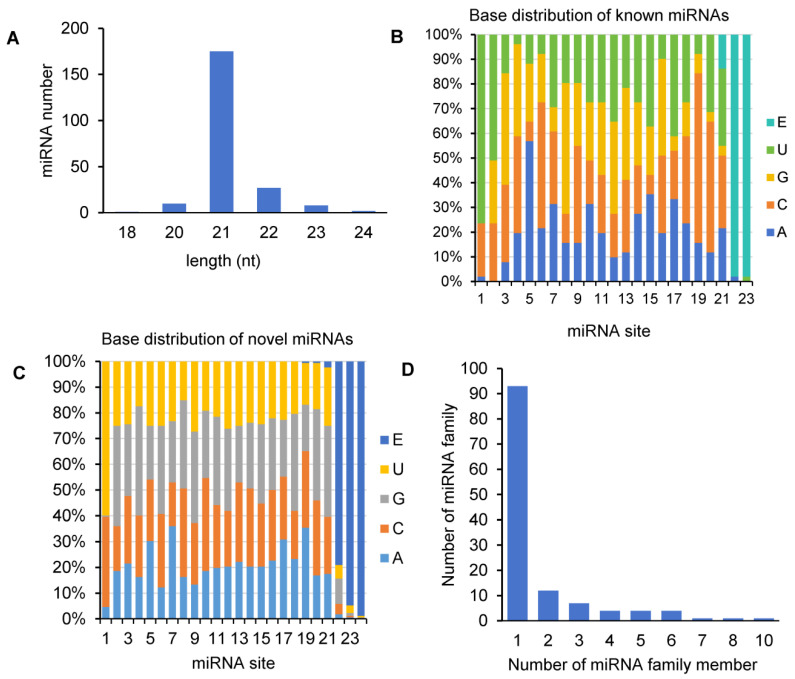
Analysis of *A. contorta* miRNAs. (**A**) Size distribution of miRNAs. (**B**) Percentage of known miRNAs starting with an A, C, G, and U, respectively. E represents empty. (**C**) Percentage of novel miRNAs starting with an A, C, G, and U, respectively. (**D**) Statistics of miRNA families and miRNA family members.

Size distribution analysis showed that the majority (71.48%) of miRNAs in *A. contorta* was 21 nt in length, about 4.48% of miRNAs were 20 nt, and about 16.59% were between 22–24 nt (Figure 2A). This is consistent with the length distribution of miRNAs from other plants [43]. First-nucleotide preference analysis showed that the 24-nt miRNAs tended to start with U in *A. contorta*, the 20- and 22-nt miRNAs tended to start with U and C, and the 21-nt miRNAs tended to start with A, U, or C. These biases are likely favorable to the interaction between miRNAs and different AGO proteins [43]. In addition, uridine and cytosine were the most common nucleotides at the end of miRNAs in *A. contorta* (Figure 2B,C).

**Figure 3:**
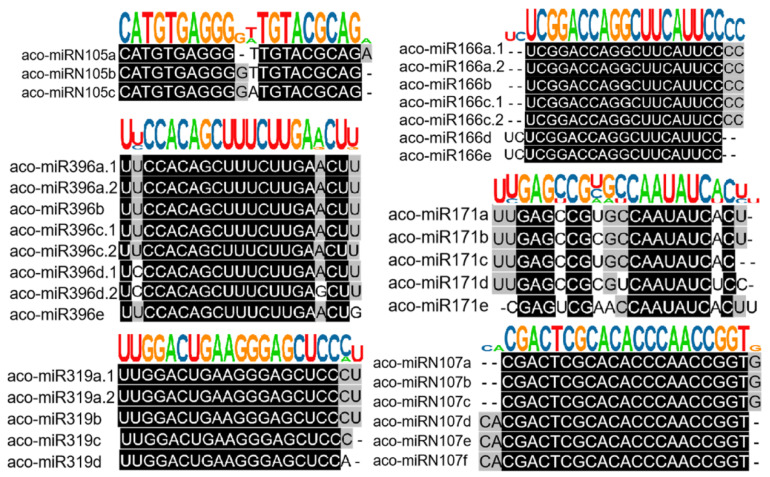
Clustering of known and novel miRNAs into families. Representative diagrams for members of both known and novel miRNA families. Sequence alignments, along with the consensus sequence logo of each family, are shown.

Based on sequence similarity, we clustered the 223 identified *A. contorta* miRNAs into families (Figure 3). Among them, the 172 novel miRNAs were clustered into 107 families, whereas the 51 known miRNAs were clustered into 20 families, including *MIR156*, *MIR159*, *MIR160*, *MIR164*, *MIR166*, *MIR168*, *MIR171*, *MIR2916*, *MIR2950*, *MIR319*, *MIR390*, *MIR394*, *MIR395*, *MIR396*, *MIR398*, *MIR399*, *MIR408*, *MIR477*, *MIR535*, and *MIR827*. The majority of the known miRNA families were deeply conserved among embryophytes [44]. The number of members in each family varied from one to ten. The majority of families consisted of only one member, followed by two and three (Figure 2D). In addition, there is a notable presence of 22-nt variants in various miRNA families, such as the three members of the *aco-MIR-Nov107* family (Figure 3).

**Figure 4:**
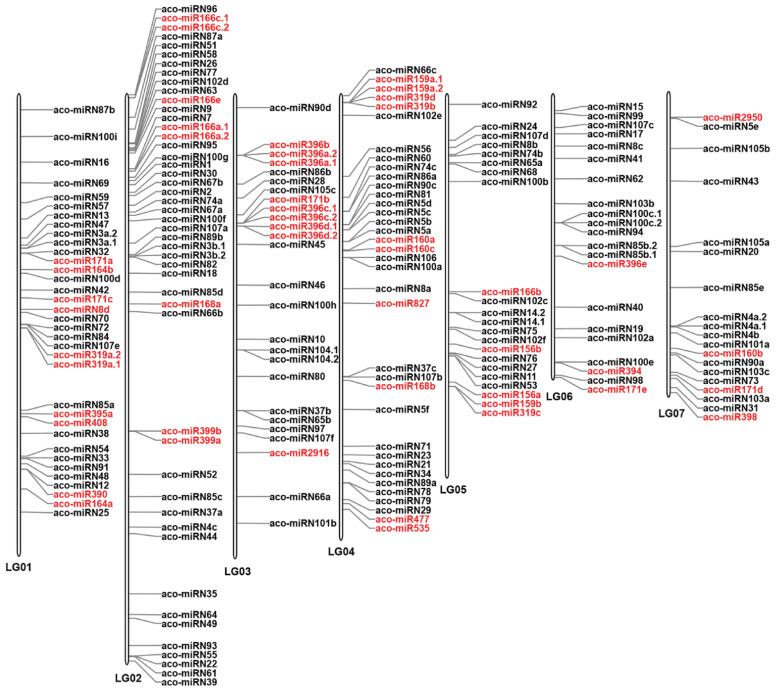
Distribution of miRNAs on seven *A. contorta* chromosomes. The position of each miRNA is marked. Known miRNAs are shown in red. Novel miRNAs are shown in black.

To investigate the distribution of miRNAs on the *A. contorta* genome, the identified miRNAs were mapped to the whole genome assembly [3]. The results showed that, of the 223 miRNAs analyzed, 210 (94.17%) were located on seven linkage groups (LGs) (Figure 4), while the rest were mapped to scaffolds. On the seven LGs, significant miRNA clusters were observed, with the majority of clusters consisting of members from the same miRNA family. In some cases, novel miRNAs were also clustered with known miRNAs. Clusters of a miRNA family could be on the same LG. They could also be located on different LGs. For instance, there were three *MIR171* family member clusters, one cluster with two members was located on LG1, and the other cluster with two members was located on LG3. The third cluster, with only one member, was located on LG7. Similarly, there were two clusters of *MIR166* family members. One cluster with five members was located on LG2. The other cluster with only one member was located on LG5. There were two clusters of *MIR396* family members. One cluster with seven members was located on LG3. The other cluster with only one member was located on LG6. Notably, seven members of the *MIR396* family and two members of the *MIR171* family were clustered together on LG3. Tandem clustering of miRNA families has been observed in other plant species, such as *Arabidopsis* and rice [30]. It indicates that the expansion of plant miRNA gene families could be primarily due to tandem and/or segmental duplication events that happened during the plant’s evolution [45,46]. Duplication of miRNA genes could result in dosage effects in regulating spatial and temporal gene expression [37].

### 2.4. Differential Expression of miRNAs

**Figure 5:**
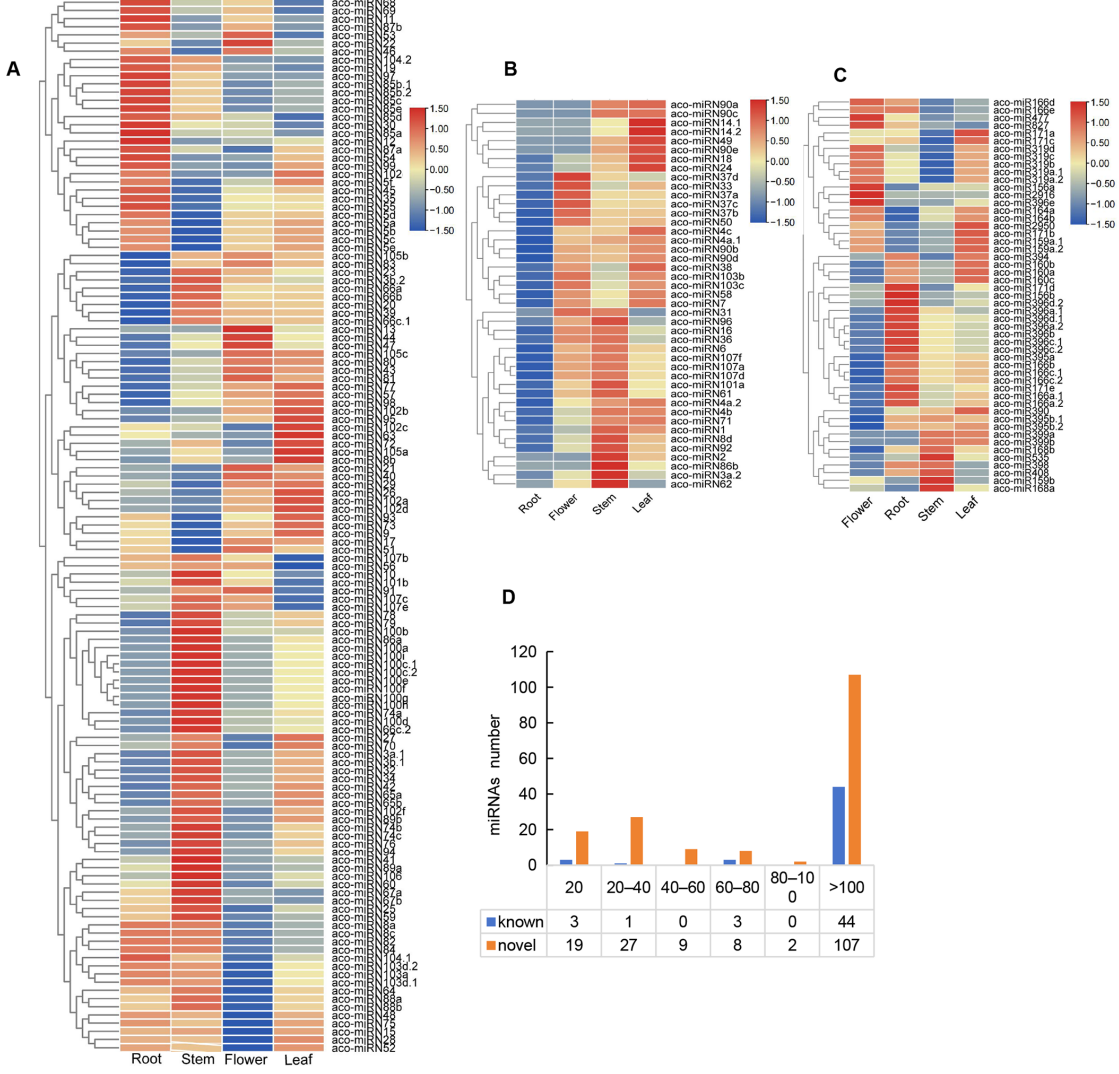
Expression of miRNAs. (**A**,**B**) Heatmap of novel miRNAs expressed in four tissues. (**C**) Heatmap of known miRNAs expressed in four tissues. (**D**) TPM distribution of miRNAs.

In order to gain deeper insights into the roles of miRNAs, we analyzed the expression profiles of 223 miRNAs in four *A. contorta* tissues (Figure 5). The results showed that the majority of miRNAs (76.68%) could be found in all of the tissues analyzed. Approximately 16.14% could be found in three tissues. The other 7.17% could be found in two tissues. Expression level analysis showed that the value of transcripts per kilobase million (TPM) for each miRNA ranged from 0 to more than 100 (Figure 5D). The majority of known (44 out of 51) and novel miRNAs (107 out of 172) were highly expressed with a TPM value of more than 100. About 11.05% of novel miRNAs were lowly expressed with a TPM value of less than 20.

Among the known miRNAs, miR171, miR159, miR2950, and miR390 exhibited a higher expression levels in leaves. MiR168, miR398, miR399, miR408, and miR535 were expressed at higher levels in stems. MiR171, miR319, and miR827 displayed higher expression levels in flowers. MiR2916 was exclusively expressed in leaves and roots. MiR156 exhibited similar expression levels in all of the tissues analyzed (Figure 5C). In addition, miR168b and miR399 were not found in roots, and miR399 was absent in flowers.

For novel miRNAs, in general, they exhibited higher expression levels in stems and lower expression levels in flowers (Figure 5A,B). Among the 172 novel miRNAs, 43 were expressed in roots, stems, and leaves. The other 129 were expressed in all of the tissues. For instance, aco-miRN41 and aco-miRN100 were highly expressed in stems. Aco-miRN12 and aco-miRN85 were abundant in roots. The levels of aco-miRN22 and aco-miRN13 were comparatively higher in flowers than in other tissues. In addition, aco-miRN14 and aco-miRN82 exhibited higher levels in leaves. Differential expression of miRNAs may be associated with their diverse functions.

### 2.5. Computational Prediction of 363 miRNA Targets

**Figure 6:**
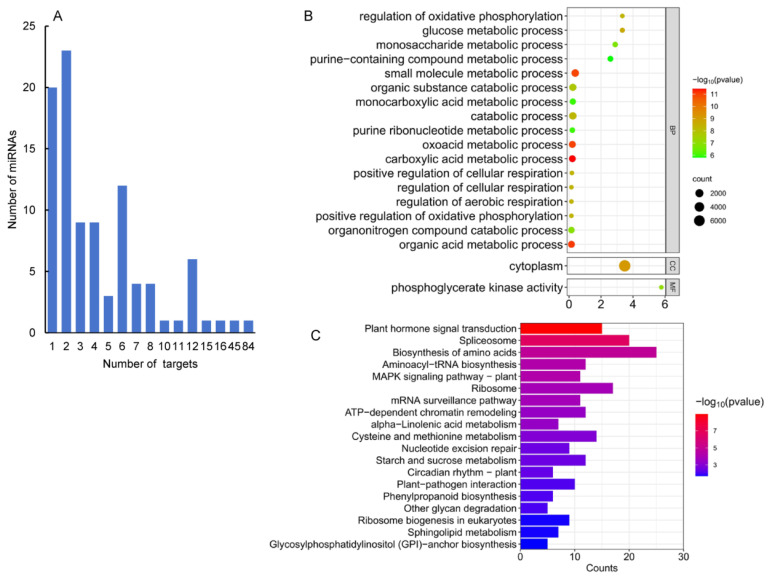
GO and KEGG analysis of predicted targets. (**A**) Number of miRNAs predicted with different numbers of targets. (**B**) Function classifications of GO terms of genes targeted by miRNAs. (**C**) KEGG enrichment analysis of genes targeted by differentially expressed miRNAs. The *X*-axis shows KEGG metabolic pathways. The *Y*-axis shows the number of targets.

MiRNAs play functions in plants mainly through cleaving the transcripts of target genes. In order to identify targets of the identified *A. contorta* miRNAs, a computational prediction was first carried out using psRNATarget [47] and CleaveLands [48]. It resulted in the identification of 363 candidate targets for 45 known and 51 novel miRNAs (Table S2). For each miRNA, the number of candidate targets most varied from one to eight (Figure 6A). Most of the target genes for known miRNAs were conserved in other plants. For instance, members of the *MIR156* family targeted genes encoding squamosa promoter-binding-like (SPL) transcription factors, which regulate plant transition from the juvenile phase to the adult phase [49]. Members of the *MIR160* family target genes encoding ARF transcription factors, which are involved in the auxin signaling pathway and play a crucial role in regulating plant growth and development [50]. Members of the *MIR159* family targeted genes encode gibberellin-regulated MYB (GAMYB) transcription factors, which are responsible for the growth of anthers, stamens, and pollen in flowering plants, such as *Arabidopsis* and *rice* [51] and might regulate the biosynthesis of flavonoids, phenolic acids, and terpenoids [14,52]. In addition, members of the *MIR164* family target genes encoding *NAC* transcription factors, members of the *MIR171* family target genes encoding Scarecrow (GRAS) transcription factors, and members of the *MIR166* family target genes encoding HB transcription factors in *A. contorta* (Table S2).

In addition, a total of 295 target genes were predicted for 51 novel miRNAs. Among them, EVM0011135.1, targeted by aco-miRN101-1, was annotated as the N-alpha-acetyltransferase gene involved in the covalent attachment of an acetyl moiety from acetyl-CoA to the free alpha-amino group at the N-terminus of a protein [53]. EVM0005856.1, targeted by aco-miRN45, is annotated as hexosaminidase involved in glycosphingolipid biosynthesis and other glycan metabolism processes [54]. Aco-miRN5 is predicted to regulate basic helix-loop-helix genes (bHLHs). Many bHLHs have been shown to be involved in plant growth and development, as well as in response to abiotic stresses, and to affect the production of secondary metabolites such as alkaloids, terpenoids, and plant classes in medicinal plants [55]. EVM0017043.1 and EVM0017043.1, targeted by aco-miRN30, are *WRKY* transcription factor genes potentially involved in the regulation of phenylpropanoid, terpenoid, and alkaloid metabolism [56]. EVM0011937.1, EVM0010138.1, EVM0005373.1, and EVM0001063.1, targeted by aco-miRN30 and aco-miRN88a, are *AP2* transcription genes potentially associated with multiple secondary metabolic processes [57].

MiRNAs play a crucial role in regulating plant growth and development by controlling the expression of target genes. To understand the regulatory function of miRNAs, it is important to determine the function of these target genes. Thus, we first performed GO enrichment analysis on 166 target genes of 50 miRNAs differentially expressed in four tissues (Figure 6B). The results showed that these targets were primarily involved in biological processes related to the carboxylic acid metabolic process, organic acid metabolic process, oxoacid metabolic process, small molecule metabolic process, and so on. It indicates that miRNAs could be involved in the regulation of metabolism in *A. contorta*. Next, KEGG pathway analysis was carried out. The results showed that the targets were mainly associated with eight pathways, including plant hormone signal transduction, spliceosome, biosynthesis of amino acids, and the MAPK signaling pathway (Figure 6C). It suggests the significance of miRNAs in *A. contorta*.

### 2.6. Validation of miRNA Targets Using Degradome Data

**Figure 7:**
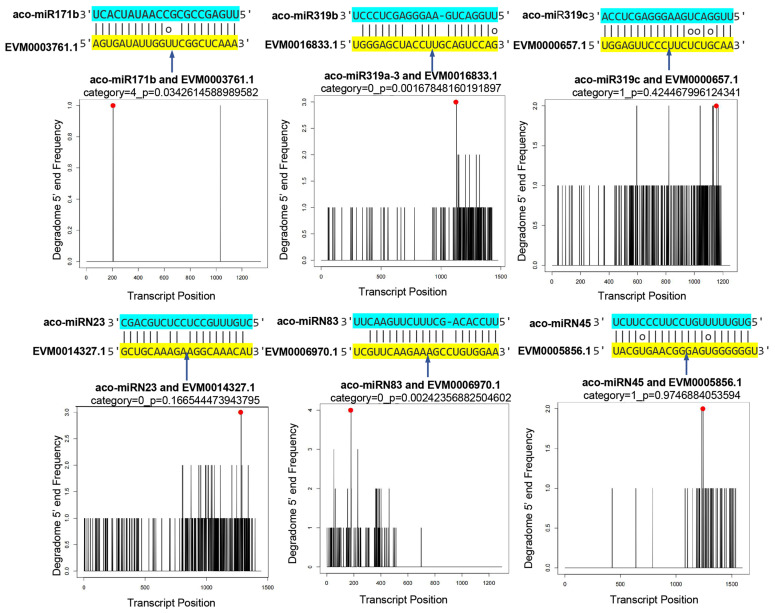
Validation of predicted mRNA targets. Cleavage positions predicted from degradome data. Red spots indicate the products resulted from miRNA-directed cleavage. Vertical arrows indicate the cleavage sites. Each top strand (blue) represents the miRNA, and each bottom strand (yellow) represents a miRNA complementary site in the target gene.2.7. Negative Correlation between the Expression of miRNAs and Their Targets.

To validate the results obtained from computational prediction using high-throughput sequencing, a degradome library was prepared and sequenced. A total of 26,317,348 clean reads, each 47 nt in length, were generated. The reads were mapped to the genome and Rfam database to filter irrelevant reads. The clean degradome data were used to analyze the cleavage of potential miRNA targets. The results showed clear cleavage signatures for 217 miRNAs analyzed, with high confidence values. Based on CleaveLand results, a total of 529 degradation sites were identified. They were classified into five types, including “0”, “1”, “2”, “3”, and “4” [48]. Some miRNAs and their targets are shown in Figure 7 and Figure S1.

**Figure 8:**
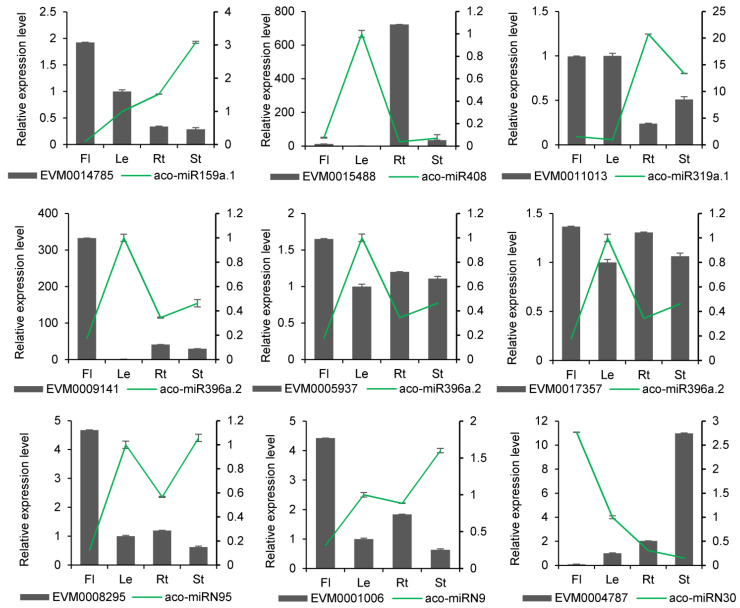
qRT-PCR validation of miRNA expression obtained through sRNAome analysis. The results from qRT-PCR and sRNAome analyses are shown in the bar graphs and line graphs, respectively. Transcript levels in leaves were arbitrarily set to 1, and the levels in other tissues were given relative to this. Data are means ± SD from three biological replicates.

In terms of molecular mechanisms of action, miRNAs typically exert their effects by degrading genes. It suggests that there might be quantitative relationships between a miRNA and its targets. Thus, we determined the expression levels of miRNAs and their targets using qRT-PCR. The results showed that miRNAs and their targets could be detected at various expression levels in different tissues. A total of 12 miRNAs and 16 target genes exhibited negative correlations in their expression profiles (Figure 8 and Figure S2). For instance, aco-miR159a.1 was highly expressed in flowers, where its target gene EVM0014785 had the lowest expression. Conversely, aco-miR159 had the lowest expression in stems, where EVM0014785 had the highest expression. This trend was also observed in other known and novel miRNA-target pairs. For example, aco-miR396e showed the highest expression in leaves, where their targets EVM0017357, EVM0009141, EVM0003367, and EVM0005937 had the lowest expression. Novel miRNA aco-miRN73 and their target also exhibited opposite expressions in four tissues. A negative correlation between miRNAs and their targets was also observed in other plant species [24]. The results suggest that the predicted miRNA targets could be authentic.

### 2.7. Identification of 441 NATs and 560 NAT-ST Pairs

**Figure 9:**
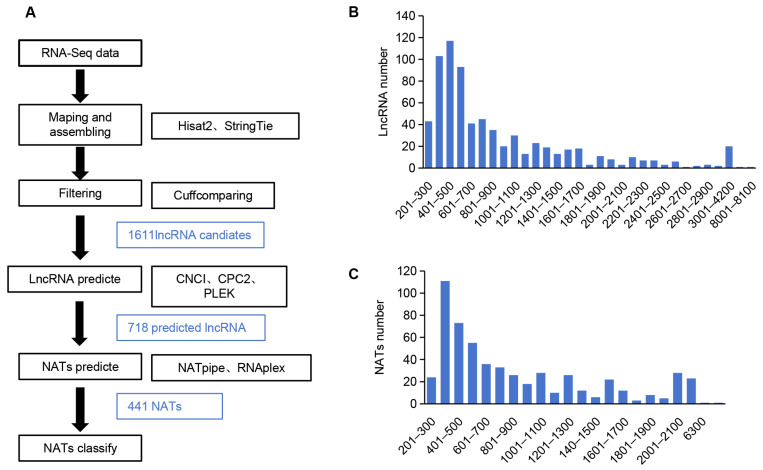
Characterization of *A. contorta* NATs. (**A**) The bioinformatics pipeline used to identify NATs in *A. contorta*. (**B**,**C**) Length distribution of lncRNAs and NATs, respectively.

NATs are natural antisense transcripts transcribed from the opposite strand of a protein-coding gene or another lncRNA. They are usually long non-coding RNAs, which play a significant regulatory role in plants through RNA–RNA interaction, long-range chromatin interaction, or by serving as miRNA decoys or small RNA precursors. To identify NATs in *A. contorta*, we first identified lncRNAs from RNA-seq clean data using Hisat2 [58], Stringtie [59], and Cufflinks [60]. A total of 1611 candidates were found. Then, we filtered the candidates using CNCI [61], CPC2 [62], and PLEK [63], resulting in the identification of 718 lncRNAs (Figure 9A and Table S3). The length distribution of the identified lncRNAs ranged from 200 to 8006 nt, with a peak at 301–600 nt (Figure 9B and Table S3) and an average length of 883 nt. Further analysis of lncRNAs using NATpipe [64] identified 441 NATs, of which 437 were classified into *cis*-NATs, and 4 were classified into *trans*-NATs based on the origin of transcripts on the genomic loci (Table S4). The length of NATs ranged from 201 to 8005 nt, with a peak at 301–400 nt (Figure 9C and Table S4).

**Figure 10:**
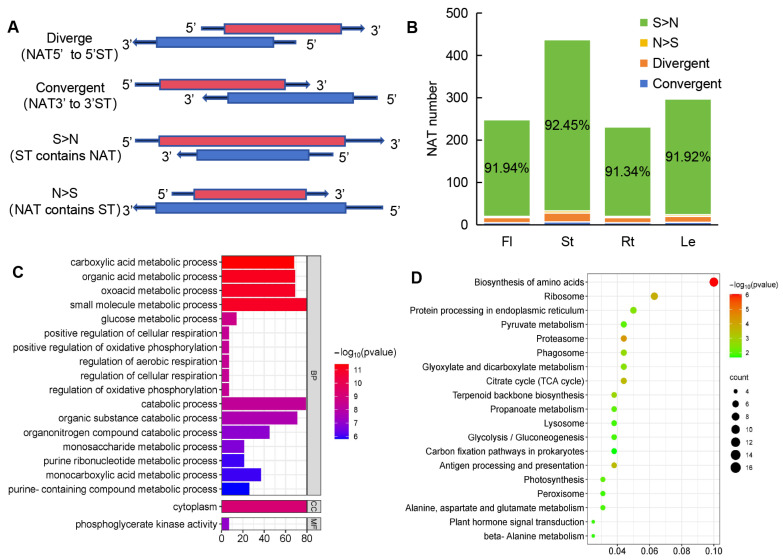
Analysis of NAT-ST pairs. (**A**) Schematic diagram of the four classes of NATs. (**B**) The number of NATs in each class. (**C**) GO enrichment of STs. (**D**) KEGG enrichment of STs.

NATs have the potential to regulate the expression of sense transcripts (STs), so NAT-ST pairs were scanned throughout the whole genome assembly of *A. contorta* using NATpipe [64]. A total of 560 NAT-ST pairs were identified (Table S5). Based on the direction and location of the sense and antisense transcripts, the 560 identified NAT-ST pairs of *cis*-NATs could be classified into four types, including convergent (with 3′-ends overlapping), divergent (with 5′-ends overlapping), STs containing NATs (S > N), and NATs containing STs (N > S) (Figure 10A), with the pair number of 23, 9, 513, and 5, respectively (Figure 10B). The predominant type was STs containing NATs, which is consistent with the results from *S. miltiorrhiza* [50].

### 2.8. Expression and Functional Enrichment Analyses of NAT-STs

**Figure 11:**
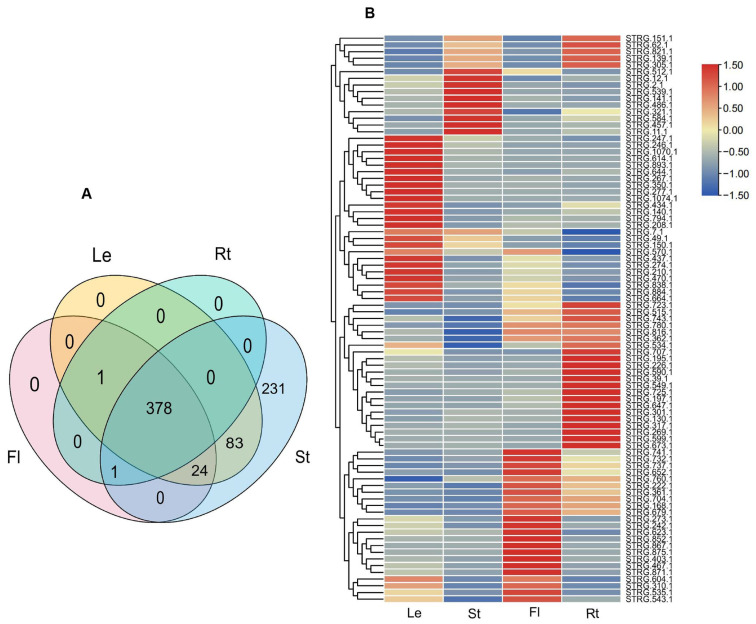
Expression of lncRNAs and NATs. (**A**) Venn diagram of differentially expressed lncRNAs. (**B**) Heatmap of differentially expression NATs.

To gain preliminary knowledge on the function of NATs, the expression of NATs in roots, stems, leaves, and flowers were analyzed using twelve RNA-Seq data from *A. contorta* Bunge, including SRR14978989, SRR14978990, SRR14978991, and SRR14978992. Among the 437 *cis*-NATs analyzed, 230 were expressed in four tissues, 18 in three tissues, 50 in two tissues, and 139 in only one tissue. Among the 230 NATs expressed in four tissues, 86 were differentially expressed, of which 25 were predominantly expressed in leaves, 24 in flowers, 27 in roots, and 10 in stems (Figure 11A,B).

To further investigate the putative function of NATs, Gene Ontology (GO) enrichment analysis was performed on STs of NATs. GO annotation showed that they were associated with various metabolic processes, such as carboxylic acid metabolic process, small molecule metabolic process, oxoacid metabolic process, and organic acid metabolic process (Figure 10C). Further, KEGG enrichment analysis showed that the sense genes were mainly involved in biological processes, such as carbon metabolism, amino acid biosynthesis, proteasome, ribosome, and citrate cycle (Figure 10D).

### 2.9. Identification of 18 Functionally Significant miRNA-NAT-ST Modules

**Figure 12:**
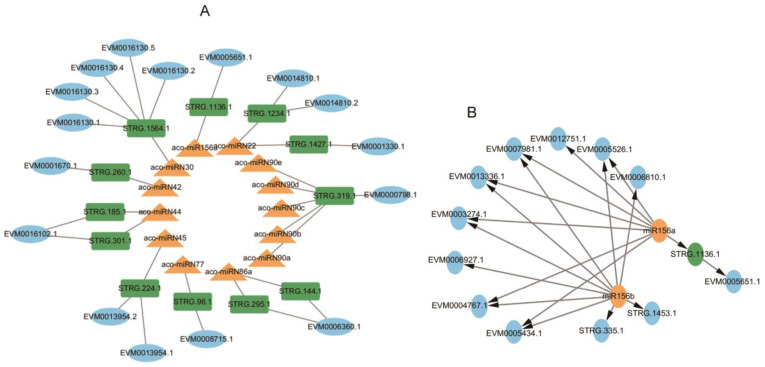
Networks of miRNA-NAT-STs. (**A**) The regulatory network of miRNA-NAT-STs. (**B**) The regulatory network of miR156. STs, miRNAs, and NATs are shown in blue, yellow, and green, respectively.

Searching NATs targeted by miRNAs using psRNATarget [47] identified 12 NATs for 13 miRNAs, forming 18 miRNA-NAT-ST modules (Figure 12A, Tables S6 and S7). Among them, the aco-miR156a-STRG.1136.1-EVM0005651.1 module could play a role in nucleocytoplasmic transport [65]. The STRG.1136.1-EVM0005651.1 pair belong to the S > N type (Figure 12A). EVM0005651.1 contains an importin repeat associated with intracellular trafficking and secretion and a RIX1 domain involved in RNA processing and ribosome assembly. In addition, the aco-miRN45-STRG.224.1-EVM0013954.1/EVM0013954.2 module could be involved in multiple physiological processes by regulating acid-base homeostasis, since EVM0013954.1 and EVM0013954.2 were identified as HCO_3_-transporter [66]. Aco-miRN42-STRG.260.1-EVM0001670.1, which encodes alpha-aminoadipic semialdehyde synthase involved in the lysine degradation pathway and can influence the conversion of aminoadipate 6-semialdehyde to diaminohexanoic acid, a key step in the biosynthesis of certain alkaloids derived from ornithine, lysine, and nicotinic acid. These results revealed the complex interaction network among miRNA, target genes, and NAT in intracellular signaling and metabolic pathways.

### 2.10. Gene Regulatory Networks for Secondary Metabolism in A. contorta

**Figure 13:**
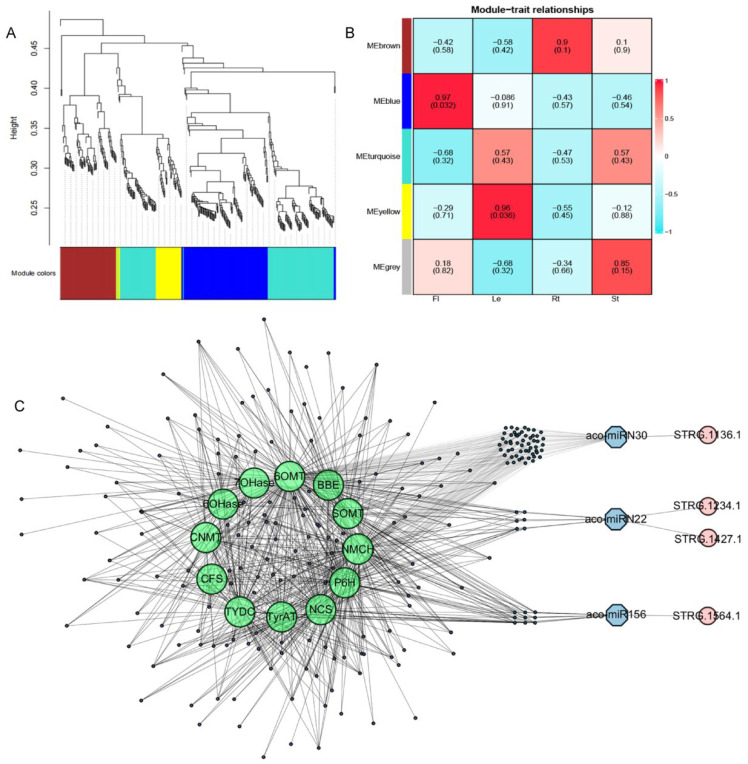
WGCNA analysis of miRNA, mRNAs, and secondary metabolism enzyme genes. (**A**) The modules of WGCNA analysis. (**B**) The relationship of modules and traits. (**C**) The mRNA-miRNA-NAT network of targets and secondary metabolic genes. Green represents genes involved in secondary metabolic pathways, blue represents miRNAs, and pink represents NATs.

AAs, aporphine alkaloids, and 1-benzylisoquinoline alkaloids (1-BIAs) are three classes of important secondary compounds produced in *A. contorta* [3,4]. Previous studies showed that a total of 91 candidate genes could be involved in their biosynthetic pathways [3,4]. To elucidate the regulatory mechanisms of enzyme genes involved in these processes, the relationship between 546 miRNA targets and 91 enzyme genes was analyzed using the WGCNA method based on RNA-seq data obtained previously [3]. After data cleaning and module-net construction, 367 miRNA target genes were found to be included in five modules, with 116 in blue, 74 in brown, 1 in grey, 138 in turquoise, and 38 in yellow modules. Among them, the blue module positively correlated with flowers, yellow module with leaves, and brown modules with roots. In addition, the yellow module showed an extremely high negative correlation with stems. Genes in the modules were selected as hub genes for further analysis. The results showed that various miRNA target genes exited in the same modules with the enzyme genes involved in AA, aporphine alkaloid, and 1-BIA biosynthesis, such as P6H, CFS, NCS, CNMT, 6OMT, TYDC, TyrAT, NMCH, 6OHase, 7OHase, SOMT, and BBE [3,4], and formed a complex regulatory network (Figure 13). These miRNA target genes mainly encoded transcription factors, protein kinases, and transporters (Table S8). It indicated that miRNAs could be involved in the regulation of AAs, aporphine alkaloids, and 1-BIAs through enzyme gene transcriptional regulation, protein phosphorylation, and metabolite transportation.

NATs are a group of lncRNAs playing significant regulatory roles in plants through various mechanisms, such as transcriptional interference, RNA-RNA interactions, and RNA-DNA interactions. Among the 560 NAT-ST pairs identified, various STs could be involved in the biosynthesis of secondary metabolites. For instance, EVM0001079.1, the ST of NAT STRG.1514.1, encodes a peroxidase, and EVM0018192.1, the ST of NAT STRG.752.1, encodes a caffeoyl-CoA-O-methyltransferase, all of which are associated with phenolic compound metabolism. The results indicated that, in *A. contorta*, the regulation of secondary metabolism, particularly the metabolism of phenolic compounds, was participated by the interaction of NATs and STs.

Previous studies showed that miRNAs could regulate NAT expression through direct cleavage. Conversely, NATs could affect miRNA levels by acting as miRNA sponges [67]. For example, wheat lncRNA35557 functioned as a competing endogenous RNA to modulate *TaNAC018* expression by acting as a decoy target for tae-miR6206 [68]. Cotton lncRNA354 had a potential binding site for miR160b, which regulated the expression of *GhARF17/18* involved in auxin signaling [69]. Among the 13 miRNAs with NAT targets, aco-miR156a also cleaved the transcripts of multiple SPL transcription genes co-expressed with enzyme genes involved in AA, aporphine alkaloid, and 1-BIA biosynthesis. Aco-miRN22 also targeted protein kinase genes co-expressed with the enzyme genes, and aco-miRN30 also potentially targeted the transcripts of *MYB* and other genes co-expressed with the enzyme genes (Figure 13C). It indicates that the NATs could play a role as miRNA sponges, which negatively regulated the level of miRNAs involved in cleaving the targets associated with secondary metabolism.

## 3. Materials and Methods

### 3.1. Plant Materials and Total RNA Extraction

Roots, stems, leaves, and flowers of *A. contorta* Bunge were collected from the Medicinal Botanical Garden located at the Institute of Medicinal Plant Development. A total of 12 samples were collected, with three biological replicates for each tissue. Sample collection was conducted at 10:00 a.m. in the field, followed by immediate freezing in liquid nitrogen. Total RNA was extracted using TRIzol (Invitrogen, Carlsbad, CA, USA). RNA integrity, purity, and concentration were assessed using an Agilent Bioanalyzer 2100 system (Agilent Technologies, Santa Clara, CA, USA), 1% agarose gel, and a NanoPhotometer^®^ spectrophotometer (IMPLEN, Westlake Village, CA, USA), respectively.

### 3.2. Small RNA Library Construction and Sequencing

Approximately 3 μg of high-quality total RNA from each sample was used for small RNA library construction. Total RNA was first purified by polyacrylamide gel electrophoresis to obtain 18–30 nt small RNAs. Then, the 5′ and 3′ adaptors were ligated to the 5′ and 3′ ends of purified small RNAs, respectively, followed by RT-PCR amplification and PCR product purification. Small RNA sequencing was performed using the HiSeq2500 sequencing platform. After sequencing, reads with low sequencing quality, 5′ end connector contamination, and reads without a 3′ end connector sequence were removed from raw sequence data using FastQC filtering and Hisat2 assembly as described previously [10].

### 3.3. Identification and Characterization of miRNAs

Clean data were mapped to the genome assembly of *A. contorta* using psRobot [38]. and miRDeep2 [39] to obtain potential miRNA, which were then aligned to the miRBase22.1 database (http://www.mirbase.org/ (accessed on 25 October 2022)) [31] for the identification of known miRNAs using BLAST [70]. Reads that could be mapped to the genome but could not be mapped to miRBase were used for novel miRNA prediction.

MiRNA targets were computationally predicted using psRNATarget [47] with the default parameters. The expression level of each identified miRNA was calculated as transcripts per million (TPM) using the formula: mapped read count/total reads × 1,000,000 [71]. To assess the expression changes between tissues, three biological replicates were pooled based on the mean TPM values. A heatmap was generated using TBtools [72] with the fold change values. For differential expression analysis of miRNAs in four tissues, egdeR was employed with the screening criteria of |log2(FC)| ≥ 1.00 and *p*-value < 0.05 [73].

### 3.4. Identification and Characterization of lncRNA and NATs

RNA-seq clean data were mapped to genome using Hisat2 [58] after quality control (QC) and removed redundancy. The assembled transcripts were generated using Stringtie. Candidate lncRNAs were selected based on specific criteria by Cufflinks, including FPKM ≥ 0.5, coverage > 1, and length > 200.

NATs were detected by aligning predicted *A. contorta* cDNA sequences to each other. If a pair of overlapping genes was matched at opposite strands with an *E*-value ≤ 1 × 10^−9^, then they were defined as a NAT-ST pair. The NAT-ST pair was located on the *A. contorta* genome to identify *cis-* and *trans*-NATs. If a pair of NAT-ST was located at the same genome locus, they were considered a *cis*-NAT-ST pair. If they were located at different genomic loci, they were considered a *trans*-NAT-ST pair. Based on the overlap between the two transcripts, *cis*-NATs were categorized into four types: convergent (3′-ends overlap), divergent (5′-ends overlap), and enclosed (full overlap), STs containing NATs (S > N), and NATs containing STs (N > S).

### 3.5. Gene Ontology and KEGG Pathway Enrichment

Go (Gene Ontology) and KEGG (Kyoto Encyclopedia of Genes and Genomes) analyses were performed using Blast2GO [74] and KOBAS 3.0 [75]. To ensure reliability, a threshold of *p*-value < 0.05 was applied for the selection of GO categories and KEGG pathways [76]. R packages topGO and clusterProfiler were used to visualize the results [77].

### 3.6. Degradome Library Construction and Analysis

Total RNA was extracted from the mixture of roots, stems, leaves, and flowers in *A. contorta* Bunge. MRNA was captured using magnetic beads. After 5′ adaptor ligation, mRNA was reversely transcribed with the biotinylated random primers, and then PCR amplified. The constructed libraries were sequenced using the Illumina Hiseq 2500 platform. After sequencing, raw tags were removed and low-quality sequences were filtered. Clean tags were clustered and aligned to the whole genome assembly of *A. contorta* for analysis of tag distribution on the genome [3]. Degradation site analysis was carried out using CleaveLand4 with *p*-value < 1 [48]. Targets were classified into five categories (0, 1, 2, 3, or 4). GO mapping was performed for the best homologs against the Gene Ontology database [78]. The identified targets were further assigned to biochemical pathways using KEGG [79].

### 3.7. Quantitative Real-Rime Reverse Transcription-PCR (qRT-PCR)

For target gene expression, 1 μg RNA was reversely transcribed into first strand cDNA using Superscript III reverse transcriptase (Invitrogen, Carlsbad, CA, USA). qRT-PCR analysis was performed on cDNAs from flowers, leaves, stems, and roots. Gene-specific primers were designed using the IDT designing primers tool (http://www.idtdna.com/scitools/Applications/RealTimePCR/ (accessed on 12 July 2023)). The expected length of amplicons ranged from 80 bp to 200 bp. *AcActin* was utilized as an internal control [3]. The expression of miRNAs was analyzed using the poly(A) adaptor RT-PCR method [80]. Real-time PCR was conducted using the Bio-Rad CFX96 Real-Time PCR Detection System. 5.8S rRNA was chosen as a reference. Gene expression data from three biological replicates were normalized.

### 3.8. WGCNA Analysis

WGCNA were analyzed using TBtools plugin WGCNA shiny [72]. The counts of miRNA targets and the alkaloid metabolism pathway genes were used as the import for data cleaning. Parameters for soft-thresholding screening and network scale-free identification were as follows: 0.8 for R^2^ cutoff and 6 for power value. For module building, mini module size was 14, module cuttree height was 0.2, and max blocksize was 5000. For hubgenes filtered, the cutoff of absolute value of kME and GS was 0.5.

## 4. Conclusions

*A. contorta* is a medicinally and academically important plant species [3]. No WGD event happened in this plant after the angiosperm-wide WGD, and it is a useful species in resolving the phylogenetic positions of magnoliids [3]. *A. contorta* has been a very important medicinal plant with various pharmacological activities. Due to the nephrotoxity and carcinogenicity of AAs, this plant has attracted widespread attention. Nowadays, scientists are making great efforts to increase its bioactive compounds and reduce or completely remove those toxic compounds. In view of these aspects, elucidating the regulatory mechanism of *A. contorta* growth, development, and metabolism is significant. In this work, 51 known and 172 novel miRNAs were found in *A. contorta*. A total of 363 targets were characterized for these miRNAs. In addition, we identified 441 *A. contorta* NATs and 560 NAT-ST pairs. Among them, 12 NATs were targets of 13 miRNAs, forming 18 miRNA-NAT-ST modules. It suggests the existence of a complex gene regulatory network in *A. contorta*. Interestingly, various miRNAs and NATs could potentially regulate secondary metabolism through the modes of miRNA-target gene–enzyme gene, NAT-ST, and NAT-miRNA-target gene–enzyme gene. It suggested the complexity of gene regulatory networks in *A. contorta*. These results enhance our understanding of intricate transcriptional regulation mechanisms and lay a solid foundation for increasing bioactive compound content and reducing toxic compound content in *A. contorta*.

## Figures and Tables

**Table 1:** Statistics of high-throughput sequencing results of *A. contorta*’s small RNA.

	Roots	Stems	Leaves	Flowers
Raw reads	27,955,448	22,405,111	19,120,948	20,509,711
Low quality	0	0	0	0
Containing ‘N’ reads	0	0	0	0
Length < 18	5,114,294	4,310,512	2,715,516	857,776
Length > 30	2,901,226	1,225,852	1,544,090	4,952,904
Clean reads	19,939,928	16,868,747	14,861,342	14,699,031
Q30 (%)	95.56	96.41	96.22	95.77

## Data Availability

The data are available in the article and its Supplementary Data.

## Supplementary Materials

The following supporting information can be downloaded at https://www.mdpi.com/article/10.3390/ijms25116043/s1.
