# Supplementary material for: Genome-Wide Identification and Characterization of miRNAs and Natural Antisense Transcripts Show the Complexity of Gene Regulatory Networks for Secondary Metabolism in Aristolochia contorta
Source: Int J Mol Sci. 2024 May 30;25(11):6043. doi: 10.3390/ijms25116043 (PMC11172604; doi:10.3390/ijms25116043)
Supplement: Supplementary file 1 [file ijms-25-06043-s001.zip › Supplemental Figures.pdf]

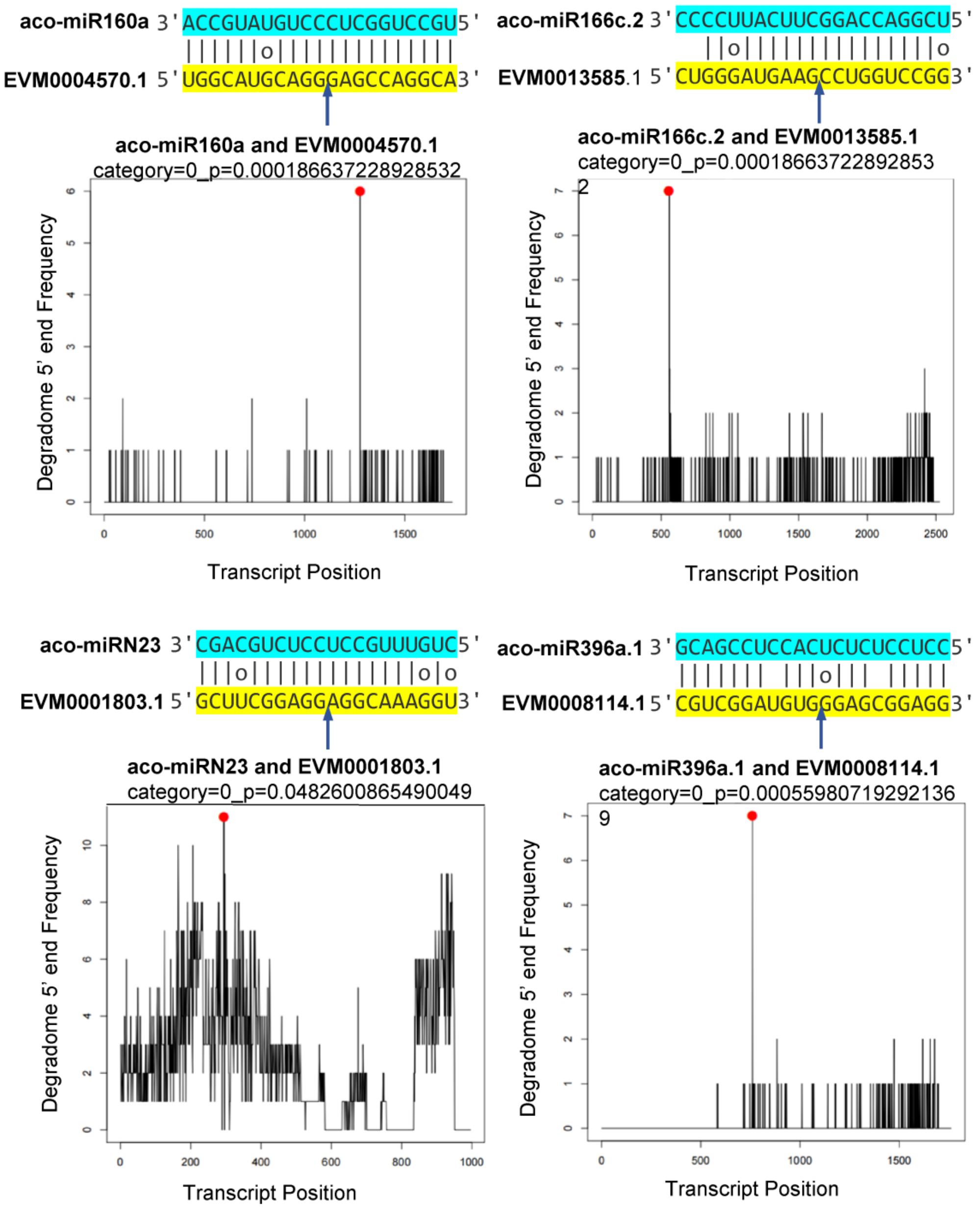

Figure S1. Validation of predicted mRNA targets. Cleavage positions predicted from degradome data. Red spots indicate the products results from miRNA-directed cleavage. Vertical arrows indicate the cleavage sites.

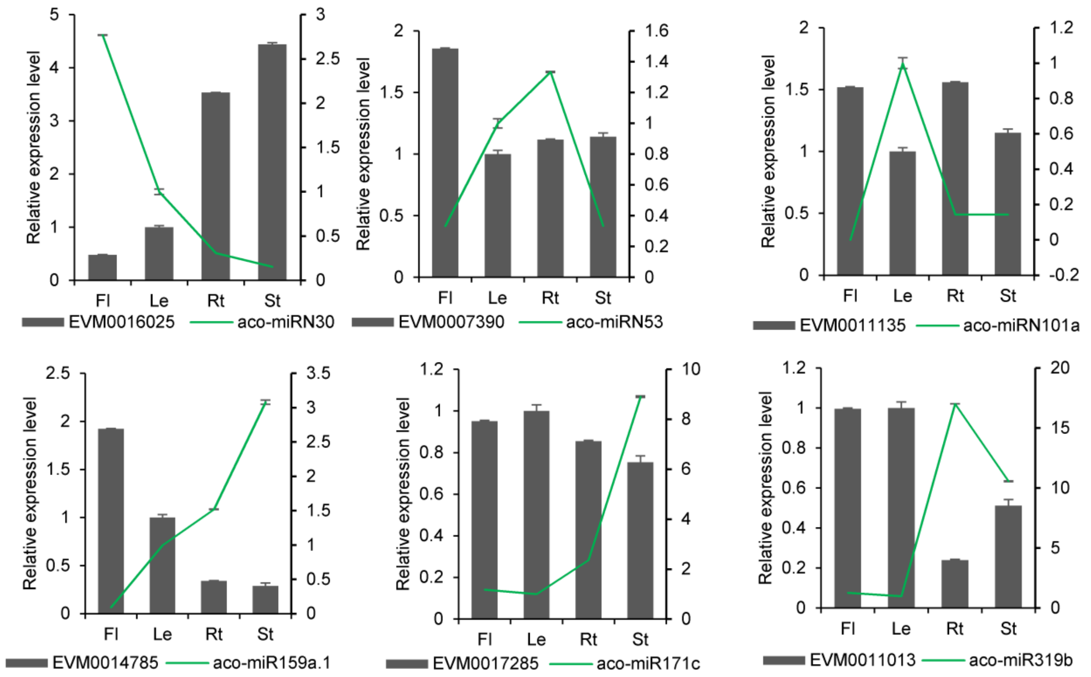

Figure S2. qRT-PCR validation of miRNA expression from sRNAome analysis. The results from qRT-PCR and sRNAome analyses are shown in bar graphs and line graphs, respectively. Transcript levels in leaves were arbitrarily set to 1 and the levels in other tissues were given relative to this. Data are means ± SD from three biological replicates.
